# The Systematic Development of a Mobile Phone Delivered Text-Messaging Tobacco Cessation Intervention in India

**DOI:** 10.1093/ntr/ntae306

**Published:** 2024-12-21

**Authors:** Miriam Sequeira, Felix Naughton, Richard Velleman, Leena Gaikwad, Pratima Murthy, Marimilha Grace Pacheco, Joseline D’souza, Ganga Nair, Rachit Shah, Seema Sambari, Urvita Bhatia, Abhijit Nadkarni

**Affiliations:** Addictions and related-Research Group, Sangath, Porvorim, Goa, India; Behavioural and Implementation Science Group, University of East Anglia, Norwich, UK; Addictions and related-Research Group, Sangath, Porvorim, Goa, India; Department of Psychology, University of Bath, Bath, UK; Bridge Medical Consultants Pvt Ltd, New Delhi, India; Department of Psychiatry, National Institute of Mental Health and Neurosciences, Bengaluru, Karnataka, India; Addictions and related-Research Group, Sangath, Porvorim, Goa, India; Addictions and related-Research Group, Sangath, Porvorim, Goa, India; Addictions and related-Research Group, Sangath, Porvorim, Goa, India; Addictions and related-Research Group, Sangath, Porvorim, Goa, India; Addictions and related-Research Group, Sangath, Porvorim, Goa, India; Addictions and related-Research Group, Sangath, Porvorim, Goa, India; Department of Psychology, Health and Professional Development, Oxford-Brookes University, Oxford, UK; Addictions and related-Research Group, Sangath, Porvorim, Goa, India; Department of Population Health, London School of Hygiene and Tropical Medicine, London, UK

## Abstract

**Introduction:**

Tobacco consumption is a leading cause of mortality globally. Eighty percent of these deaths occur in low- and middle-income countries. Despite this, there is a large treatment gap due to both demand and supply-side barriers. Digital interventions are an innovative solution to bridge this gap. We describe the systematic development of ToQuit, a text-messaging intervention for tobacco cessation in India.

**Aims and Methods:**

ToQuit was developed in sequential steps: (1) Identifying Behaviour Change Techniques (BCTs) from evidence-based tobacco cessation interventions; (2) Identifying additional BCTs through in-depth interviews; (3) Online expert survey to rate BCTs on feasibility, acceptability, and perceived effectiveness when delivered via text messaging; and (4) A consultation workshop with practitioners.

**Results:**

Thirty BCTs were identified from steps 1 and 2. Three were excluded in step 3. The final intervention included 27 BCTs delivered over three phases: phase 1—orientation (information about antecedents and consequences of tobacco consumption, goal setting, reattribution, pros and cons of quitting); phase 2—skill building (self-monitoring, avoidance of cues for behavior, behavior substitution, distraction, stress management, handling urges, restructuring physical and social environments, instructions on how to perform a behavior, prompts and cues, problem-solving, social support, and referrals among others); and phase 3—relapse prevention (psychoeducation about lapse and relapse, review goals, action planning, and feedback on behavior). The intervention is delivered via text messages over 8 weeks with 2–3 messages a day, 2–3 days a week.

**Conclusions:**

ToQuit is a contextually relevant and potentially scalable text-messaging intervention for tobacco cessation in resource-constrained settings.

**Implications:**

This manuscript is an important piece in the development of contextually relevant digital interventions for the cessation of both smoked and smokeless forms of tobacco. The detailed description of the steps followed in developing the ToQuit intervention will help others replicate these procedures while developing similar interventions responsive to their contexts; particularly in low- and middle-income countries.

## Introduction

Every year 6 million people globally die from tobacco-use-related problems, and almost 1 million of these deaths occur in India.^1^ India is the second largest consumer (266.8 million adults)^2^ and third largest producer of tobacco in the world.^3^ Unlike most other countries, smokeless tobacco is the preferred mode of tobacco consumption in India (21% of smokeless tobacco-using adults vs. 11% of adults who smoke).^2^ Ninety-two percent of this tobacco-using population has been estimated to have not received an effective tobacco cessation intervention in their lifetime.^4^ A key reason for this is the shortage of trained healthcare workers and a lack of tobacco cessation interventions provided outside of healthcare settings; hence non-resource-intensive interventions are needed in resource-constrained settings such as India and other low- and middle-income countries.^5^

The Government of India’s existing text-messaging tobacco cessation intervention^6^ was adapted from the World Health Organisation’s (WHO) “Be He@lthy, Be Mobile” tobacco cessation handbook.^7^ However, details of the adaptation such as the nature of these consultations and collaboration with stakeholders, methods used, adaptations made to the Indian context, and their rationale are not publicly available. More importantly, users of tobacco were not actively engaged in the intervention development. Finally, although the effectiveness of other individual and community-level tobacco cessation interventions has been tested in India^8–11^ they are either designed for only people who smoke^8,10^ or as workplace interventions and not individual-level interventions for the general population.^9,11^

Our study addressed these gaps by systematically developing ToQuit, a contextually relevant and potentially scalable text-messaging intervention for tobacco cessation. In this paper, we describe the processes that led to the development of ToQuit, beginning with the identification of potentially promising Behavioural Change Techniques (BCTs) for tobacco cessation and ending with the final content of the ToQuit intervention and the conceptual framework laying out the hypothesized pathways to behavior change.

We followed Michie et al.’s^12^ theory-based taxonomy to identify BCTs which defines and categorizes BCTs based on their functions: those that directly address the capability and motivation of the individual or create opportunities to engage in the desired behavior. Although this method was originally developed to standardize BCTs and explain the “active ingredients” used in behavioral interventions for smoking cessation, it has been applied to other types of behavioral interventions as well.^13^

## Materials and Methods

### Setting

The study was conducted in Goa (population of 1.4 million) in western India. The prevalence of current tobacco use in Goa is 9.7% (2.6% women, 18.2% men).^2^ Goa has two Tobacco Cessation Centres (TCCs) located in district hospitals. Cessation counselors and social workers are available at these centers six days a week. In addition, eight Primary Health Centres (PHCs) have cessation services twice a month.^14^

### Study Design

Our intervention development process used a mixed methods approach which included four sequential steps described below.

#### Step 1: Examination of the Existing Relevant Evidence Base

In the absence of a review of digital interventions for all forms of tobacco use at the time of developing this intervention, in accordance with the Medical Research Council’s guide to developing complex interventions^15^ we identified the most recent, relevant (mobile-based intervention), and high-quality (robust and reproducible methodology) systematic review of smoking cessation interventions.^16^ This review included randomized, quasi-randomized, and cluster-randomized trials with participants (age >13 years) who were smokers at the time of enrollment into the study. The review included smoking cessation interventions delivered via text messaging or smartphone apps. Interventions where mobile phones were adjuncts to face-to-face or internet programs were excluded. The primary outcome was smoking abstinence at the longest follow-up, and at least 6 months from baseline. Where multiple measures were available, we preferred sustained abstinence to point prevalence abstinence, and biochemically validated results to self-report. Additional details about the methods followed in the review are summarized in Appendix 1.

Two researchers independently extracted intervention-related data from only text-message-based smoking cessation interventions mentioned in the review. These data were then synthesized by a third researcher using content analysis. Content analysis is a qualitative research method that helps with coding textual data into themes or categories to identify common patterns, content, or relationships across studies.^17^ In addition, we reviewed three WHO^18–20^ and two Indian evidence-based tobacco cessation manuals^21,22^ to supplement the smoking cessation strategies with strategies for other forms of tobacco use. We identified specific intervention techniques that were described in each study and mapped them onto the BCT taxonomy.^12^

#### Step 2: In-Depth Interviews With Stakeholders

This step aimed to (1) Identify the patterns of tobacco use in the local context; (2) Examine the content and delivery mode of BCTs that experts and intended recipients might perceive as useful; (3) Explore and define treatment expectations and desired outcomes for people using tobacco; and (4) Use data from these interviews to define intervention content, delivery, and recruitment processes. A detailed report of this step has been published separately.^23^

We conducted in-depth semi-structured interviews with the following stakeholders:

(a) Two categories of experts namely, (i) National tobacco cessation experts such as directors of National Tobacco Control Programmes (NTCP) and (ii) dentists trained to provide counseling at TCCs in Goa.
(b) Adult (≥18 years) current users (at least once in the past 28 days) of tobacco in Goa.

Recruitment and data collection:

We identified experts through our existing academic connections who had expertise in tobacco cessation practice (purposive sampling) and sent them emails eliciting their interest to participate in the study. From those who replied expressing interest in participation, informed consent was obtained in-person or via email, and interviews were conducted either in-person or remotely (video conference or telephone) depending on their geographical location. At the end of their interview, we asked them to recommend others (snowball sampling) whom they deemed appropriate for participation in this study. All interviews with experts were conducted in English by trained researchers (GN and RS), audio recorded, and then transcribed.

We recruited current users of tobacco among patients attending the TCC at Goa’s tertiary care dental hospital and through health screening camps at workplaces. Patients waiting at the clinic for their appointment were universally approached by a trained qualitative researcher (JD, MGP, or SS) who explained the project objectives to them in their preferred language and screened for study eligibility.

At the workplaces, we conducted a seminar on “Health and Wellbeing” as a precursor to screening to minimize the potential stigmatization from screening for tobacco use. After the session, we individually screened all those who participated in the seminar. The inclusion criteria for this sample were: being ≥18 years of age and having used tobacco in any form at least once in the past 28 days. Those who used tobacco but not in the past 28 days or spoke a language that was not comprehensible to the research team were excluded from the study.

In both settings, we took interested and eligible prospective participants to a private room within the premises and gave them time to read the information sheet about the research study. For those who preferred, the researcher read it aloud for them. After addressing their questions, we obtained their written informed consent and collected their sociodemographic details and current tobacco use-related data. The researcher then completed the interview (~60 minutes) in the participant’s preferred language. All interviews were audio recorded and later transcribed and translated into English for analysis. We stopped data collection once we reached data saturation, defined as no new information being discovered in data analysis, which signals redundancy.^24^

The research objectives of the study and literature review findings informed the interview guides in Appendix 2. GN developed the first version of the interview guide which was reviewed by the principal investigator. The revised version was tested within the team and probes were added wherever necessary. Finally, the guides were revised after completing a third of the interviews to simplify the language and prioritize questions that we needed more information about. We asked users of tobacco about their own patterns of tobacco use, reasons for initiation and sustained tobacco use, the impact of tobacco use on various aspects of their lives, motivators, methods to quit and outcomes of those methods, experiences and preferences regarding mobile phone features and content areas of the proposed intervention. We asked practitioners about their patients’ patterns of tobacco use, the intervention techniques they used, and the factors affecting treatment uptake. For both sets of participants, we also presented sample BCT content areas and asked them to comment on the perceived importance and utility of each of those.

Two independent coders (GN and MS for the expert dataset; JD and MGP for the tobacco user dataset) analyzed the data using N-Vivo version 11. We used a thematic analysis approach to identify intervention content areas from the transcripts.^25^ The analysis involved the generation of codes from raw data, and then deriving themes by retrieving pieces of data pertaining to codes and examining their meaning in relation to the research questions.

#### Step 3: Online Expert Survey

We created a consolidated list of all BCTs identified from steps 1 and 2. We then converted these BCTs into an online survey on Qualtrics to elicit information on the acceptability (likelihood of the strategy being accepted by users of tobacco), feasibility (suitability for delivery via text messages), and perceived effectiveness of each when delivered via text message. We tested a draft version of the survey within the team for accuracy of content, ease of use, and understanding of backend data reports. We launched the survey after making minor changes to the language and sent it via email to international tobacco cessation experts identified from manuscripts referenced in the systematic review, an internet search of international tobacco research centers, personal networks of the project investigators, and the membership database of the International Network for Brief Interventions and Other Drugs (INEBRIA). We did not collect sociodemographic data from the respondents to minimize the time taken to complete the survey. Consenting participants received a definition of each BCT and an example of how it might be implemented in a tobacco cessation intervention. They were then asked to rate each BCT on a 5-point Likert scale (1—strongly disagree to 5—strongly agree) separately for acceptability, feasibility, and perceived effectiveness. They were also provided an option to give qualitative feedback. We calculated the mean scores for each BCT on acceptability, feasibility, and perceived effectiveness. BCTs with a score of 3.5 and above on each domain moved on to the next step of intervention development considering that a score of 3 on the Likert scale stood for “neither agree nor disagree.” Scores above 3 indicated that the BCT was endorsed (“agree”) by the experts for this specific intervention.

#### Step 4: Synthesis of Findings to Inform Intervention Content

We then took the BCTs which made it through step 3 into a content development workshop with local tobacco cessation practitioners comprising psychologists, psychiatrists, and a TCC counselor selected through purposive sampling. All had experience of working with users of tobacco from the local community. The aims of the workshop were to: (1) develop the conceptual framework of the intervention using the outputs of the formative research, and (2) understand the cultural acceptability and clinical relevance of each BCT in the context of a text-messaging intervention. After participants were introduced to BCT definitions and implementation examples, they were divided into two groups. We asked each group to (1) Examine the list of BCTs; (2) Check whether they wanted to add/exclude BCTs to/from that list and provide the rationale for doing so; (3) Merge BCTs that could form a single component; (4) Organize the selected components into a conceptual framework for the ToQuit intervention; and (5) Describe the hypothesized mechanisms of action of the intervention.

Each group then presented their conceptual frameworks; after which the two frameworks were merged into a single framework through consensus building. Participants then suggested the sequence and frequency of text message delivery for the intervention. After the workshop, we developed individual text messages for each of the intervention components and organized the delivery as per the sequence and frequency suggested by the workshop participants.

### Ethics

This study was approved by the Institutional Review Boards of the host institution, Sangath (AN_2018_36), the recruitment site, that is, state-level Dental College and Hospital, and the Indian Council for Medical Research (2018-0161). All tobacco-using participants were offered an information leaflet about the harmful effects of tobacco and a brief intervention based on the WHO’s 5As and 5R^20^ delivered by trained researchers.

## Results

### Step 1: Examination of the Existing Evidence

We found 26 studies from the most recent and relevant Cochrane review on mobile phone text-messaging and app-based smoking cessation interventions.^16^ Fifteen of the 26 studies were text-messaging interventions, some supplemented with telephone counseling, and the rest were app-based. Additionally, we examined WHO’s Toolkit for Delivering the 5A’s and 5R’s Brief Tobacco Interventions in Primary Care,^20^ WHO’s Quit Guide for Tobacco Users,^18^ Starting Tobacco Cessation Services by the National Institute of Mental Health and Neurosciences, India,^22^ and National Guidelines for Tobacco Cessation by the Government of India.^21^

We identified a total of 30 distinct BCTs from all these sources (Table 1). BCTs included (1) behavioral techniques such as avoidance of cues, restructuring the social and physical environment, distraction, and substitution; (2) cognitive techniques such as self-talk, thinking about the pros and cons of using tobacco and comparative imagining of future outcomes; and (3) other techniques such as social support. The application of these BCTs in a text message intervention for tobacco cessation is provided in Appendix 3.

**Table 1.** BCTs Identified From Step 1 in the Intervention Development Process

Name of BCT	Description of BCT as per the taxonomy
Action planning	Prompt detailed planning of the performance of the behavior.
Avoidance/reducing exposure to cues for behavior	Advise on how to avoid exposure to specific social and contextual/physical cues for the behavior, including changing daily or weekly routines.
Behavior practice/rehearsal	Prompt practice or rehearsal of the performance of the behavior one or more times in a context or at a time when the performance may not be necessary, to increase habit and skill.
Behavior substitution	Prompt substitution of the unwanted behavior with a wanted or neutral behavior.
Body changes	Alter body structure, functioning, or support directly to facilitate behavior change.
Comparative imagining of future outcomes	Prompt or advise the imagining and comparing of future outcomes of changed vs. unchanged behavior.
Distraction	Advise or arrange to use an alternative focus for attention to avoid triggers for unwanted behavior.
Goal setting (behavior)	Set a goal defined in terms of a positive outcome of wanted behavior.
Habit reversal	Prompt rehearsal and repetition of an alternative behavior to replace an unwanted habitual behavior.
Information about antecedents	Provide information about antecedents that reliably predict the performance of the behavior.
Information about health consequences.	Provide information about the health consequences of performing the behavior.
Information about social and environmental consequences	Provide information about the social and environmental consequences of performing the behavior.
Pharmacological support	Provide, or encourage the use of or adherence to, drugs to facilitate behavior change.
Problem-solving	Analyze, or prompt the person to analyze, factors influencing the behavior and generate or select strategies that include overcoming barriers and/or increasing facilitators.
Prompts and cues	Introduce or define environmental or social stimulus with the purpose of prompting or cueing the behavior.
Pros and cons	Advise the person to identify and compare reasons for wanting (pros) and not wanting to (cons) change the behavior.
Re-attribution	Elicit perceived causes of behavior and suggest alternative explanations.
Reduce negative emotions	Advise on ways of reducing negative emotions to facilitate the performance of the behavior.
Restructuring the physical environment	Change, or advise to change the physical environment to facilitate the performance of the wanted behavior or create barriers to the unwanted behavior.
Restructuring the social environment	Change, or advise to change the social environment to facilitate the performance of the wanted behavior or create barriers to the unwanted behavior.
Self-monitoring of behavior	Establish a method for the person to monitor and record their behavior as part of a behavior change strategy.
Self-talk	Prompt positive self-talk (aloud or silently) before and during the behavior.
Social support (emotional)	Advise on, arrange, or provide emotional social support for the performance of the behavior.
Social support (practical)	Advise on, arrange, or provide practical help for the performance of the behavior.
Social support (unspecified)	Advise on, arrange, or provide social support or noncontingent praise or reward for the performance of the behavior.
Verbal persuasion about capability	Tell the person that they can successfully perform the wanted behavior, arguing against self-doubts, and asserting that they can and will succeed.
Instruction on how to perform the behavior	Advise or agree on how to perform the behavior.
Feedback on behavior	Monitor and provide evaluative feedback on the performance of the behavior.
Commitment	Eliciting a commitment statement on quitting.
Review outcome goal	Review outcome goal(s) jointly with the person and consider modifying goal(s) in light of achievement.

BCTs = Behaviour Change Techniques.

### Step 2: In-Depth Interviews

We conducted semi-structured in-depth interviews with 23 users of tobacco (22 males and 1 female; 7 smoked tobacco, 13 used smokeless tobacco, and 3 used both types) and 13 national tobacco cessation experts and practitioners (4 males and 9 females). The mean age of the users was 39.5 years (range 23–65 years). The majority of users were married and had secondary-level education. Eighteen were recruited from the dental college TCC and five were recruited from workplaces. The average years of experience of the practitioners was 20.4 years (0–23 years), respectively, with areas of specialization ranging from periodontics, psychiatry, psychology, oncology, surgery, and pharmacy. See Appendix 4 for more details about the participants.

Users had already tried a range of techniques to quit tobacco including motivating self-talk, removing reminders of tobacco from their physical environment, pharmacotherapy, substitution with other addictive or nonaddictive substances, avoiding triggers, Nicotine Replacement Therapy, and indigenous medicines, among others. Key themes from the interviews with tobacco users and practitioners are listed in Table 2 and some excerpts from the interviews are presented in Box 1.

**Table 2.** Key Themes From the Interviews With Tobacco Users and Practitioners With Regard to the ToQuit Intervention Content and Delivery

Source	Content	Delivery
Users of tobacco	Harmful effects of tobacco on the user’s body. Negative effects of their tobacco use on their loved ones. Regular motivational and inspiring quotes. Instructions on developing a quit plan and urge management plan. Weblinks to access more information or in-person services. The tone of the messages should be motivational.	Personalization Some preferred personalized messages (ie, with the user’s name and tailored to the pattern of usage). Most users preferred generic messages to protect privacy. Some were undecided. Duration Range of preferences regarding duration; from 1 week to 1 year. Most preferred at least 2 months of messages to sustain change. Time of messages Most preferred early in the morning as that was when they most experienced the urge to use. Preferred receiving messages on alternate days. Longer messages over the weekend or in the evening post-work hours to enable focused reading and reflection. Level of engagement Half of those interviewed did not want to reply to messages. Almost all preferred SMS over Interactive Voice Response (IVR) as they could re-visit the message later.
Practitioner	Initial messages should focus only on motivation to change as in their experience intrinsic motivation was the most important factor leading to tobacco cessation and relapse was higher if the intervention did not develop intrinsic motivation to change. Messages on the health impacts of tobacco on the individual and their loved ones were the most effective motivator to change. Address comorbid mental health problems like depression and anxiety. Encourage the use of NRT only under the supervision of a practitioner. Enhance self-efficacy beliefs.	Practitioners unanimously endorsed messages personalized to the individual’s name and patterns of tobacco use (eg, match SMS timing to urge timing of participant). The suggested duration of the intervention ranged from 1 to 6 months.

NRT = Nicotine Replacement Therapy.

### Step 3: Expert Survey

**Figure 1.**
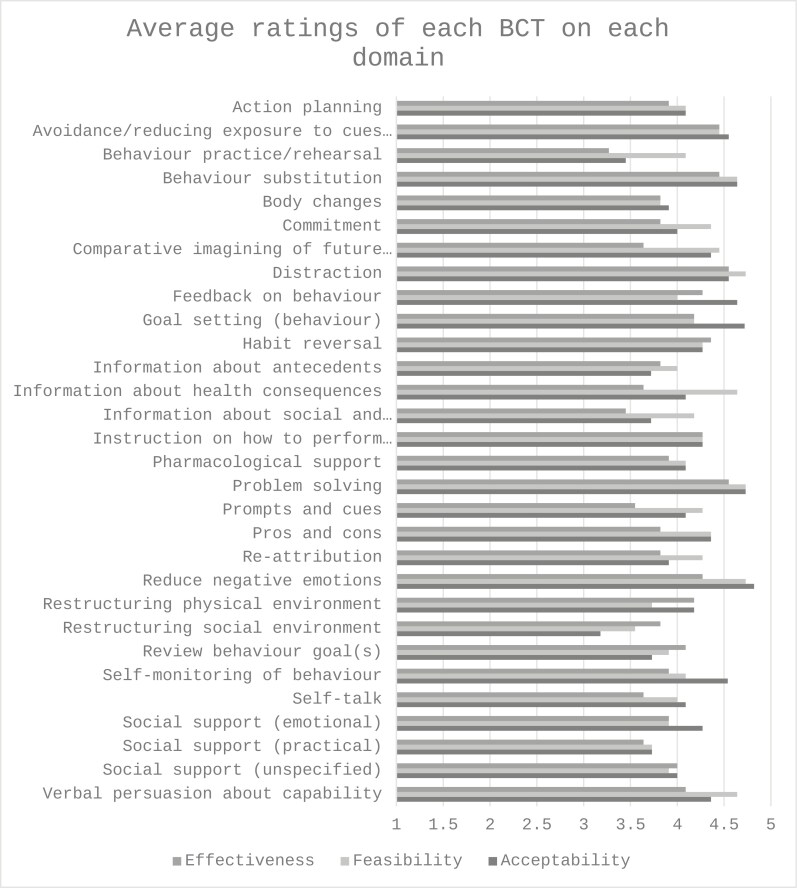
Experts’ ratings of BCTs on acceptability, feasibility, and perceived effectiveness for this intervention. BCTs = Behaviour Change Techniques.

We sent the survey to 65 experts, 22 (34%) responded and 11 (17%) completed it. Out of the 30 BCTs, three (behavioral practice, body changes, and restructuring the social environment) received an average score lower than 3.5 (out of 5) on two or more domains (acceptability, feasibility, and perceived effectiveness) and were excluded. Figure 1 illustrates these ratings. The remaining 27 BCTs were taken into step 4 of the intervention development process described below.

### Step 4: Content Development Workshops

A total of 10 participants (six psychologists, three psychiatrists, and one TCC counselor with experience of delivering tobacco cessation treatments ranging from 3 to 20 years) were divided into two groups for the workshop. Both groups chose to retain all 27 BCTs that made it to step 4 of the intervention development. Although additional BCTs were not included in the list, participants added specifics to some BCTs such as the type of information to be shared, and organized the list into phase-wise conceptual frameworks described below. Appendices 5 and 6 illustrate the conceptual frameworks of groups A and B organized into three and four phases, respectively.

**Figure 2.**
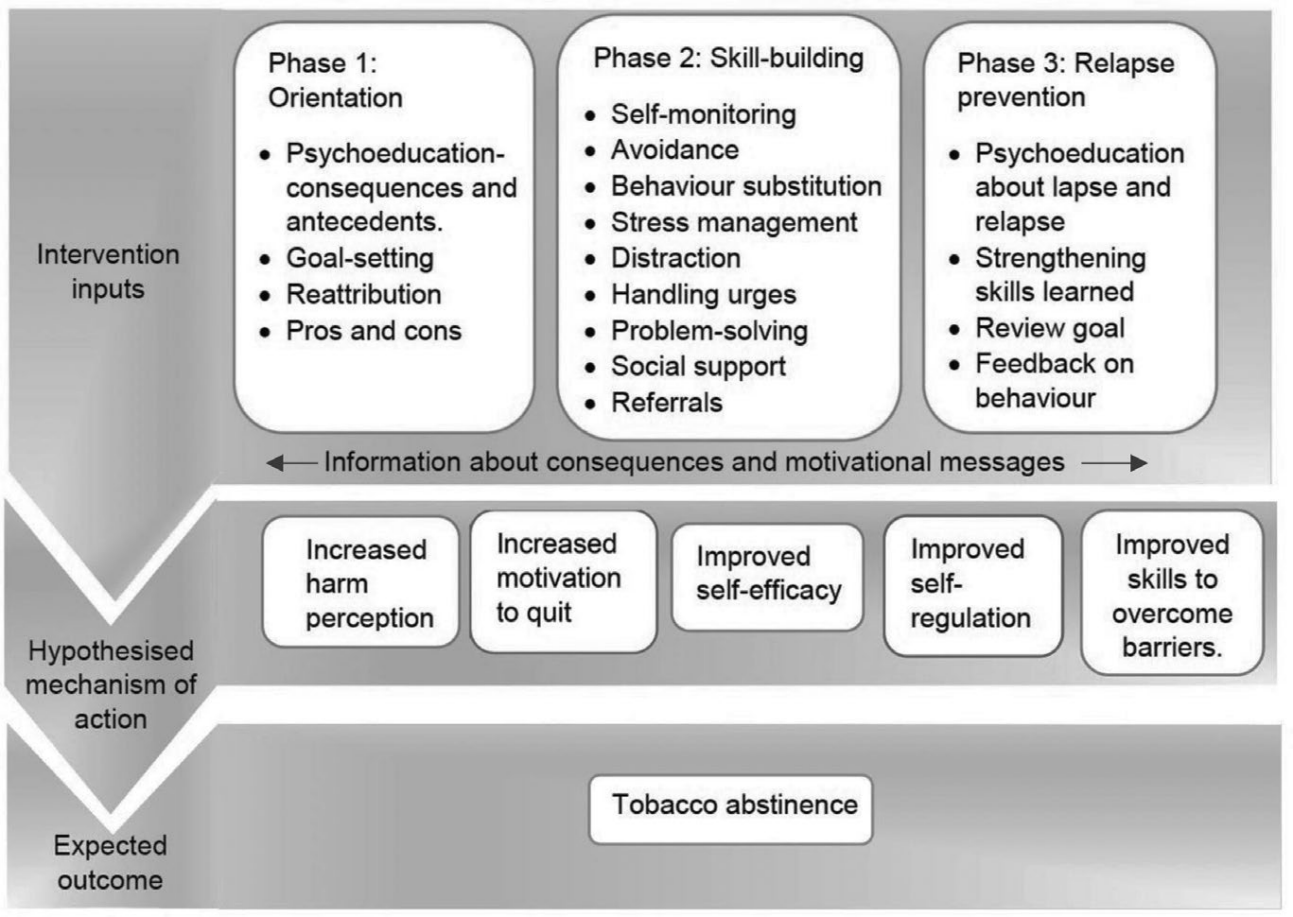
The ToQuit intervention conceptual framework.

Since the frameworks of both groups appeared very similar in their structure and aims, we were able to merge them into a single framework consisting of three phases namely orientation, skill building, and relapse prevention. The final conceptual framework for the ToQuit intervention is illustrated in Figure 2. The discussion also led to a few proposed mechanisms of action which are also reflected in Figure 2.

Restructuring the social environment, although rejected in the expert survey, was re-introduced by the workshop participants as they deemed it to be an acceptable, feasible, and effective strategy for tobacco cessation. Based on their function, we merged some BCTs into a single domain area. Information about health consequences, information about social and environmental consequences, and information about antecedents were merged as “psychoeducation about consequences and antecedents”. Restructuring the physical environment, restructuring the social environment, and avoidance/reducing exposure to cues for behavior were merged as “avoidance.” Information about antecedents, instructions on how to perform the behavior, prompts, and cues were merged as “handling urges.” The key skills delivered in phase 2 (substitution, avoidance strategies, handling urges, stress management, and social support) were merged as “strengthening skills learned.”

Participants strongly supported the need for personalized messages based on readiness to quit. They suggested sending a single message after phase 1 to gauge the readiness to proceed to the next phase. Their rationale was that it would be futile to send messages regarding strategies to quit when the person is not ready to quit. Hence, they proposed a branching logic at that stage—participants who are ready to quit get messages from phase 2 while those not yet ready to quit, continue to receive messages based on motivational enhancement. However, we did not personalize message delivery in this way as it would mean that those participants who did not respond would not receive messages that could potentially be useful. This decision was based on user response rates from our previous study which developed a text-messaging intervention for hazardous drinking.^26^

The final ToQuit intervention derived from the above-mentioned methodologies consisted of three phases described below.

#### Phase 1: Orientation

The key components of this phase are psychoeducation (eg, negative consequences on health, family, and finances) and goal setting. Users of tobacco will be oriented toward the intervention aims, frequency of messages, and duration. Although personalized goals will not be set for reasons mentioned already, information will be provided about the usefulness of goal setting and the types of goals.

#### Phase 2: Skill Building

During this phase, users of tobacco will receive messages about practical skills or techniques to use in their quit attempts. These will include avoidance strategies, behavior substitution, distraction, stress management, self-monitoring of behavior, problem-solving, and seeking social and professional support (referrals).

#### Phase 3: Relapse Prevention

The key components delivered in this phase will be information about lapse and relapse (including messages to depersonalize lapses) and the encouragement of maintenance of skills learned in phase 2.

Motivational and informational messages were sent throughout the intervention.

This conceptual framework guided the development of the structure and detailed messages for the ToQuit intervention which are delivered over 8 weeks. The structure and distribution of intervention messages over 8 weeks are detailed in Appendix 7. Informed by the qualitative interviews with tobacco users, we framed messages for thrice a week, except for the first week which includes one additional day of introductory messages which is sent on the date of enrollment. The other messages are matched to participant preferences that is, participants who preferred receiving all messages on the weekend will be sent messages on three consecutive days beginning Friday and ending on Sunday while others receive them on alternate days of the week.

## Discussion

Our paper describes the process of developing a contextually relevant evidence-based text-messaging tobacco cessation intervention for India. The ToQuit intervention that we developed at the end of this process is an attempt to address the treatment provider-related barriers by increasing access to a simple evidence-based tobacco cessation intervention delivered through text messages.

We hypothesize that the intervention components of phase 1 will increase the perception of harm from tobacco and improve motivation to quit. The components of phase 2 are designed to improve self-efficacy beliefs and self-regulation through skill building. Finally, the components of phase 3 are hypothesized to improve skills to address barriers that might arise in the future triggering relapse. Collectively, these outcomes are hypothesized to result in tobacco abstinence.

The development of this intervention demonstrated to us that despite a very clear focus on intervention components that are contextually informed, most content in the final intervention package has several commonalities with other tobacco cessation interventions delivered in person or using technology. A recent systematic review^27^ found that person-delivered BCTs were effective but found limited evidence that BCTs delivered as information pamphlets and using digital media were associated with higher smoking cessation. The exception to this was the BCT taxonomy grouping of “rewards” which predicted higher smoking cessation rates when delivered in writing concluding that this finding adds to the mixed literature on the associations between the number of BCTs delivered digitally and behavioral outcomes; with some showing positive effects and others negative. The ToQuit intervention does not include the BCT cluster on rewards due to issues with feasibility within a text-messaging intervention such as implementing the reward system. This systematic review also found that three BCTs consistently predicted higher smoking cessation rates, namely, prompting commitment, social reward, and identity associated with changed behavior. The ToQuit intervention included only the first of these BCTs as the other two were difficult to implement in the form of text messages. For example, the BCT “social reward” is defined as arranging for a verbal or nonverbal reward if and only if the desired behavior was performed. Since this is an individual unidirectional messaging intervention, it renders the effective implementation of this BCT infeasible. Future iterations of this intervention could consider innovative ways to include these BCTs to increase the effectiveness of the intervention.

Grounded in both global (step 1) and contextual evidence (steps 2–4), our intervention further strengthens the existing evidence for commonly used tobacco cessation brief interventions. Our findings from the triangulation of data from the empirical evidence and interviews with local stakeholders indicate the universal applicability of brief interventions for tobacco cessation and strengthens our confidence in how they can be generalized across cultural contexts. These findings are consistent with the experiences of other investigators outside India who have adapted psychological treatments for use in culturally diverse settings.^28^

We note some limitations of our intervention development process. The systematic review we used in step 1 was limited to smoking cessation interventions and had very few studies from low- and middle-income countries. However, to mitigate this limitation, we supplemented these data with an in-depth examination of tobacco cessation manuals used by local practitioners and qualitative interviews with local stakeholders. Second, we acknowledge the limitations related to our sample size and response rate for the expert survey and sample diversity for the qualitative interviews. However, we supplemented these sources of data with insights from practitioners working with the local community during the content development workshops. Third, two-way messaging (ie, sending personalized messages in response to participants’ replies) could increase the acceptability and effectiveness of our intervention. However, as stated earlier, our previous work^29^ in the same settings with an alcohol-related text-messaging intervention, demonstrated that participants prefer unidirectional push messages (messages being delivered without requiring input from the participant) and tend to be nonresponsive to pull messages (message delivery is dependent on an input from the participant) in line with similar studies in high income countried (HICs).^30,31^ This implied to us that introducing pull messages into the intervention would mean that many of the participants would not receive the entire intervention. Hence, although this method might limit active engagement, it allows participants to revisit the intervention content at a later stage, as opposed to receiving none or a limited dosage of the intervention if they disengage. Fourth, some critics of contextually developed interventions point out that the utility of these interventions can be overstated, as the developers assume that cultural groups are a homogenous entity. To address this gap, the intervention was initially tested with 26 participants from across the country.^32^ The intervention content and delivery were then revised based on the qualitative data gathered from post-intervention semi-structured interviews conducted with a subset (*n* = 17) of this population. The revised intervention was then tested via a feasibility randomised controlled trial (RCT) with 98 users of tobacco recruited from all over India.^33^ In addition, the advantage of our intervention framework is that it provides a general outline that can be used to tailor messages depending on the needs of cultural subgroups. Another critique of our intervention development process could be the nonalignment with an existing theoretical framework. We extracted BCTs from theory-aligned text-messaging interventions and supplemented these with local stakeholder inputs to co-develop a conceptual framework that aligns well with other theory-based text-messaging interventions.^34^ Another limitation is that although we conducted interviews in the participant’s preferred language, we did not follow the process of back-translation of the interview guide which could have increased the reliability of the tool when used across different languages. Finally, we reexperienced limitations similar to our previous m-health interventions^26,29^ that are common to resource-constrained settings like India, such as the limited reach and poor quality of mobile telecommunication network infrastructure.

In summary, in accordance with the recommended guidelines for intervention development, we have integrated the global evidence base (top–down) with the contextual experiences (bottom–up) in developing this intervention. A similar evidence-based model developed and tested in our context^35^ broadly defines sequential stages of intervention development consisting of the following stages: (1) information gathering, (2) preliminary adaptation design, (3) preliminary adaptation tests, and (4) adaptation refinement. We have described the first two stages of this process in this paper and stages three and four in others. We tested the acceptability and feasibility of ToQuit^32^ followed by a pilot RCT.^33^ ToQuit has the potential to be an inexpensive and scalable response to the increasing burden of tobacco use in settings with limited trained human resources.

## Supplementary Material

Supplementary material is available at *Nicotine and Tobacco Research* online.

Box 1: **Quotes from users from users of tobacco and cessation practitioners during the semi-structured interviews**

Impact on family as a motivator to quit tobacco use:

My wife says, ‘You put tobacco in your mouth in front of people on the road. We feel ashamed. The children also [feel ashamed].’ Then I tried many times to leave it [tobacco

[42-year-old male user of SLT].

Negative health consequences being a strong motivator to quit:

I don’t know how many of the patients that we see here really go back and do all of that [cessation advice]. To me somewhere I feel they go back, and they continue. . . - - Unless and until we find something [lesions, adhesions, etc.] in their mouth and they are scared – ‘Now what next?’ - That fear is there. Otherwise, I think they are less receptive generally.

[33-year-old female dentist at a TCC].

Frequency of the messages:

If you send it (messages) everyday then it will be irritating. Once or twice (in a week) is okay. Not when we are at work. . . then we will not pay too much attention to it. Better to send it in the evening after 7.30 pm when we are relaxed.

[62-year-old male who smokes]

ntae306_suppl_Supplementary_Appendices

## Data Availability

Data that support the findings of this study are available from the corresponding author, AN, upon reasonable request.
