# Supplementary material for: The Systematic Development of a Mobile Phone Delivered Text-Messaging Tobacco Cessation Intervention in India
Source: Nicotine Tob Res. 2024 Dec 21;27(9):1616–25. doi: 10.1093/ntr/ntae306 (PMC12370465; doi:10.1093/ntr/ntae306)
Supplement: ntae306_suppl_Supplementary_Appendices [file ntae306_suppl_supplementary_appendices.zip › ntae306_suppl_Supplementary_Appendix_3.docx]

**APPENDIX 3:** List of BCTs extracted from step 1 along with their standardised definitions and application to a tobacco cessation text messaging intervention.

**Alt text:** The table below has four columns. Column one lists out the serial number, column two – the name of the BCT, column three – the description of the BCT and column four – the potential application of that BCT to a tobacco cessation text messaging intervention.

| **S/N** | **Name of BCT** | **Description of BCT** | **Application to tobacco cessation text messaging intervention** |
| --- | --- | --- | --- |
| 1 | Action planning | Prompt detailed planning of performance of the behaviour (must include at least one of context, frequency, duration and intensity). | Prompt planning the performance of a personalised activity at a specific time each day to overcome cravings to use tobacco. |
| 2 | Avoidance/reducing exposure to cues for behaviour | Advise on how to avoid exposure to specific social and contextual/physical cues for the behaviour, including changing daily or weekly routines. | Avoidance of people, places, situations or things that trigger tobacco usage. |
| 3 | Behaviour practice/rehearsal | Prompt practice or rehearsal of the performance of the behaviour one or more times in a context or at a time when the performance may not be necessary, in order to increase habit and skill. | Provide script to practice saying ‘no’ to peers when offered tobacco. |
| 4 | Behaviour substitution | Prompt substitution of the unwanted behaviour with a wanted or neutral behaviour. | Suggest chewing gum instead of chewing tobacco. |
| 5 | Body changes | Alter body structure, functioning or support directly to facilitate behaviour change. | Link to guided relaxation exercise to focus on body changes during urges and countering them. |
| 6 | Comparative imagining of future outcomes | Prompt or advise the imagining and comparing of future outcomes of changed versus unchanged behaviour. | Suggest to calculate money saved from not using tobacco and what could be purchased with that money instead. |
| 7 | Distraction | Advise or arrange to use an alternative focus for attention to avoid triggers for unwanted behaviour. | Help person identify and engage in distraction activities when they experience the urge to use tobacco. |
| 8 | Goal setting (behaviour) | Set or agree on a goal defined in terms of a positive outcome of wanted behaviour. | Set a personalised quit date. |
| 9 | Habit reversal | Prompt rehearsal and repetition of an alternative behaviour to replace an unwanted habitual behaviour. | Suggest making it a habit to chew gum instead of tobacco. |
| 10 | Information about antecedents | Provide information about antecedents (*e.g. social and environmental situations and events, emotions, cognitions)* that reliably predict performance of the behaviour. | Ask participant to record situations/thoughts/feelings that trigger urge to use tobacco. |
| 11 | Information about health consequences. | Provide information (e.g. written, verbal, visual) about health consequences of performing the behaviour. | Inform participant about the health impacts of using tobacco. |
| 12 | Information about social and environmental consequences (including financial consequences on family) | Provide information (e.g. written, verbal, visual) about social and environmental consequences of performing the behaviour. | Ask participants to list out ways in which their tobacco use negatively impacts themselves and their loved ones in social situations. |
| 13 | Pharmacological support | Provide, or encourage the use of or adherence to, drugs to facilitate behaviour change. | Advice the person to ask their family physician for nicotine replacement therapy. |
| 14 | Problem-solving | Analyse , or prompt the person to analyse, factors influencing the behaviour and generate or select strategies that include overcoming barriers and/or increasing facilitators (includes ‘Relapse Prevention’ and ‘Coping Planning’) | Encourage the person to identify triggers that generate urges to smoke and develop strategies for avoiding these. |
| 15 | Prompts and cues | Introduce or define environmental or social stimulus with the purpose of prompting or cueing the behaviour. The prompt or cue would normally occur at the time or place of performance. | Advise the person to carry a reminder card in their wallet which lists out the reasons why they want to quit tobacco. |
| 16 | Pros and cons | Advise the person to identify and compare reasons for wanting (pros) and not wanting to (cons) change the behaviour. | Ask person to weigh the cost and benefits of quitting tobacco. |
| 17 | Re-attribution | Elicit perceived causes of behaviour and suggest alternative explanations (e.g. external or internal and stable or unstable). | Provide alternate explanation for myths. For example, If the person attributes their tobacco use as essential to comfortable bowel movements, suggest that the ‘real’ cause for difficulty with bowel movements is brought about because of the use of tobacco. |
| 18 | Reduce negative emotions | Advise on ways of reducing negative emotions to facilitate performance of the behaviour (includes ‘Stress Management’). | Provide exercises on how to manage irritation, anxiety or other negative emotions that might arise from quitting tobacco. |
| 19 | Restructuring the physical environment | Change, or advise to change the physical environment in order to facilitate performance of the wanted behaviour or create barriers to the unwanted behaviour (other than prompts/cues, rewards and punishments). | Advice the participant to change their travel routes to avoid shops that sell tobacco. |
| 20 | Restructuring the social environment | Change, or advise to change the social environment in order to facilitate performance of the wanted behaviour or create barriers to the unwanted behaviour (other than prompts/cues, rewards and punishments). | Suggest the benefits of restructuring their social network so that they interact only with non-tobacco users while attempting to quit. |
| 21 | Self-monitoring of behaviour | Establish a method for the person to monitor and record their behaviour as part of a behaviour change strategy. | Ask the participant to maintain a daily record of tobacco use behaviour. |
| 22 | Self-talk | Prompt positive self-talk (aloud or silently) before and during the behaviour. | Suggest self-motivating phrases that the participant can use as self-talk when experiencing the urge to use tobacco. |
| 23 | Social support (emotional) | Advise on, arrange, or provide emotional social support (e.g. from friends, relatives, colleagues, ‘buddies’ or staff) for performance of the behaviour. | Suggest the benefit of sharing their quit goal with a significant other and specifying the type of emotional response they would like to receive from them. |
| 24 | Social support (practical) | Advise on, arrange, or provide practical help (e.g. from friends, relatives, colleagues, ‘buddies’ or staff) for performance of the behaviour. | Advise participant to tell their partner/colleague to lead the distraction activity. |
| 25 | Social support (unspecified) | Advise on, arrange or provide social support (e.g. from friends, relatives, colleagues,’ buddies’ or staff) or non-contingent praise or reward for performance of the behaviour. It includes encouragement and counselling, but only when it is directed at the behaviour. | Provide information on tobacco cessation clinics that they could visit in person. |
| 26 | Verbal persuasion about capability | Tell the person that they can successfully  perform the wanted behaviour, arguing  against self-doubts and asserting that they can and will succeed. | Send motivational messages instilling hope and self-efficacy beliefs in the participant. |
| 27 | Instruction on how to perform the behaviour | Advise or agree on how to perform the behaviour (includes ‘Skills training’) | Advising the person to step away from the situation, sip on some water, listen to music, and remind themselves that they can handle the urge. |
| 28 | Feedback on behaviour | Monitor and provide informative or evaluative feedback on performance of the behaviour (e.g. form, frequency, duration, intensity) | Inform the person about their progress towards their quit goal. |
| 29 | Commitment | Eliciting a commitment statement on quitting | Asking participant to repeat a commitment statement. |
| 30 | Review outcome goal | Review outcome goal(s) jointly with the person and consider modifying goal(s) in light of achievement. This may lead to resetting the same goal, a small change in that goal or setting a new goal instead of, or in addition to the first. | Ask if participant was able to meet the weekly goal and suggest modifying goal if difficulty meeting the set goal. |
