# Supplementary material for: The Systematic Development of a Mobile Phone Delivered Text-Messaging Tobacco Cessation Intervention in India
Source: Nicotine Tob Res. 2024 Dec 21;27(9):1616–25. doi: 10.1093/ntr/ntae306 (PMC12370465; doi:10.1093/ntr/ntae306)
Supplement: ntae306_suppl_Supplementary_Appendices [file ntae306_suppl_supplementary_appendices.zip › ntae306_suppl_Supplementary_Appendix_4.docx]

**Appendix 4.1:** Tobacco cessation practitioner characteristics.

**Alt text:** The table below provides the socio-demographic details of tobacco cessation practitioners with whom semi-structured qualitative interviews were conducted in step two of the intervention development process.

| **Demographic characteristics** | **Tobacco cessation practitioners (N=13)**  **n (%)** |
| --- | --- |
| Gender  Male  Female | 4 (30.8%)  9 (69.2%) |
| Age (in years)  26 – 35  36 – 45  46 – 55  56 – 65 | 7 (53.8%)  2 (15.4%)  2 (15.4%)  2 (15.4%) |
| Highest educational level  MA Psychology  MD Psychiatry  MD Periodontics Surgery  MS Surgery  M Pharm  PhD | 1 (7.7%)  2 (15.4%)  7 (53.8%)  1 (7.7%)  1 (7.7%)  1 (7.7%) |
| Current designation/role  Assistant Professor  Associate Professor  Chief of Clinical services  Lecturer, Public Health Dentistry  Proprietor of Pharmacy  Professor and Head of the Department  Psychologist  Senior Psychiatrist | 1 (7.7%)  1 (7.7%)  1 (7.7%)  5 (38.5%)  1 (7.7%)  1 (7.7%)  1 (7.7%)  1 (7.7%)  1 (7.7%) |
| Years in practice  0 – 5  6 – 10  11 – 15  16 – 20  20 + | 6 (46.1%)  2 (15.4%)  1 (7.7%)  2 (15.4%)  2 (15.4%) |

**Appendix 4.2:** Users of tobacco characteristics

**Alt text:** The table below provides the socio-demographic details of users of tobacco with whom semi-structured qualitative interviews were conducted in step two of the intervention development process.

| **Demographic characteristics** | **Tobacco users (N=23)**  **n (%)** |
| --- | --- |
| Gender  Male  Female | 22 (95.7%)  1 (4.3%) |
| Type of tobacco used  Smoked  Smokeless  Both | 7 (30.4%)  13 (56.6%)  3 (13.0%) |
| Age (in years)  21 – 30  31 – 40  41 – 50  51 – 60  61 – 70 | 8 (34.7%)  5 (21.7%)  4 (17.3%)  4 (17.3%)  2 (8.6%) |
| Marital status  Single  Married | 5 (21.7%)  18 (78.3%) |
| Highest educational level  No formal schooling  Primary  Secondary  Higher secondary  Graduate  Postgraduate | 1 (4.3%)  6 (26.1%)  11 (47.8%)  1 (4.3%)  2 (8.7%)  2 (8.7%) |
| Employment status  Employed full time  Employed part time  Unemployed  Retired  Employed on daily wage-basis | 16 (69.6%)  1 (4.3%)  4 (17.4%)  1 (4.3%)  1 (4.3%) |
