# Supplementary material for: The Systematic Development of a Mobile Phone Delivered Text-Messaging Tobacco Cessation Intervention in India
Source: Nicotine Tob Res. 2024 Dec 21;27(9):1616–25. doi: 10.1093/ntr/ntae306 (PMC12370465; doi:10.1093/ntr/ntae306)
Supplement: ntae306_suppl_Supplementary_Appendices [file ntae306_suppl_supplementary_appendices.zip › ntae306_suppl_Supplementary_Appendix_5.docx]

**Appendix 5: Group A’s conceptual framework for the ToQuit intervention.**

Keep it up!

Verbal persuasion about capability (i.e., motivational messages)

How?

Why?
