# Supplementary material for: The Systematic Development of a Mobile Phone Delivered Text-Messaging Tobacco Cessation Intervention in India
Source: Nicotine Tob Res. 2024 Dec 21;27(9):1616–25. doi: 10.1093/ntr/ntae306 (PMC12370465; doi:10.1093/ntr/ntae306)
Supplement: ntae306_suppl_Supplementary_Appendices [file ntae306_suppl_supplementary_appendices.zip › ntae306_suppl_Supplementary_Appendix_6.docx]

**Appendix 6:** Group B’s conceptual framework for the ToQuit intervention.

**Alt text:** The figure below has four boxes for four of the different phases that Group B categorised the BCTs into during the intervention development workshop.
