# Supplementary material for: The Systematic Development of a Mobile Phone Delivered Text-Messaging Tobacco Cessation Intervention in India
Source: Nicotine Tob Res. 2024 Dec 21;27(9):1616–25. doi: 10.1093/ntr/ntae306 (PMC12370465; doi:10.1093/ntr/ntae306)
Supplement: ntae306_suppl_Supplementary_Appendices [file ntae306_suppl_supplementary_appendices.zip › ntae306_suppl_Supplementary_Appendix_7.docx]

**Appendix 7: The ToQuit intervention structure and message delivery schedule.**

| Phases | Week | Theme |  | Description | BCTs |
| --- | --- | --- | --- | --- | --- |
| 1: Orientation | Week1 | PSYCHOEDUCATION ABOUT CONSEQUENCES | Day1 | Introduction to ToQuit | Not applicable |
|  |  |  | Day2 | Psychoeducation about consequences | Information about health consequences |
|  |  |  | Day3 | Benefits of quitting | Pros and cons of quitting |
|  |  |  | Day4 | Psychoeducation continued | Information about social and financial consequences Information about emotional consequences |
|  | Week2 | GOAL SETTING | Day1 | Defining goal setting | Goal setting (outcome) |
|  |  |  | Day2 | Encourage personal goal setting | Prompt commitment |
|  |  |  | Day3 | Managing goals | Goal setting (behaviour) |
| 2: Skill-building | Week3 | BEHAVIOURAL STRATEGIES | Day1 | Self-monitoring of behaviour | Self-monitoring of behaviour |
|  |  | Reminder about quit date | Day2 | Identifying and avoiding triggers | Information about antecedents Avoidance of cues to behaviour Restructuring the physical environment |
|  |  |  | Day3 | Substitution + Distraction | Behaviour substitution Distraction |
|  | Week4 | COGNITIVE STRATEGIES | Day1 | Self-talk | Self-talk |
|  |  | Reminder about quit date | Day2 | Stress-management | Reduce negative emotions |
|  |  |  | Day3 | Imagining future outcomes | Comparative imagining of future outcomes |
|  | Week5 | ADDITIONAL SUPPORT | Day1 | Benefits of seeking social support | Social support (emotional) |
|  |  | Reminder about quit date | Day2 | Information about pharmacological support | Pharmacological support |
|  |  |  | Day3 | Referrals to tobacco cessation clinics | Social support (unspecified) |
|  | Week6 | HANDLING URGES/CRAVINGS | Day1 | Identifying personal urges/cravings | Information about antecedents Self-monitoring of behaviour |
|  |  | Reminder about quit date | Day2 | Managing cravings | Instruction on how to perform the behaviour (handling urges) |
|  |  |  | Day3 | Problem-solving | Problem-solving + Action planning |
| 3: Relapse prevention | Week7 | RELAPSE PREVENTION | Day1 | Understanding lapse and relapse | Information |
|  |  |  | Day2 | Relapse prevention strategies | Action planning |
|  |  |  | Day3 | Review benefits of quitting | Pros and cons + Prompts and cues |
|  | Week8 | MAINTENANCE | Day1 | Review strategies |  |
|  |  |  | Day2 | Review progress |  |
|  |  |  | Day3 | Feedback | Feedback on behaviour |
