# Supplementary material for: The Systematic Development of a Mobile Phone Delivered Text-Messaging Tobacco Cessation Intervention in India
Source: Nicotine Tob Res. 2024 Dec 21;27(9):1616–25. doi: 10.1093/ntr/ntae306 (PMC12370465; doi:10.1093/ntr/ntae306)
Supplement: ntae306_suppl_Supplementary_Appendices [file ntae306_suppl_supplementary_appendices.zip › ntae306_suppl_Supplementary_Appendix_1.docx]

**Appendix 1:** Summary of the methods followed by the systematic review titled ‘Mobile phone text messaging and app‐based interventions for smoking cessation’ by Whittaker and colleagues, 2019.

**Alt text:** A bullet point summary of the methods followed by the systematic review titled ‘Mobile phone text messaging and app‐based interventions for smoking cessation’ by Whittaker and colleagues published in 2019. It mentions the types of studies, populations, interventions, comparison groups and outcomes included in the review and details about the search methods, data collection, analysis, extraction and risk of bias assessment methods followed.

**Types of studies**

Randomised or quasi‐randomised trials. Cluster‐randomised trials were eligible for inclusion.

**Types of participants**

People who smoked at study enrolment.

**Types of interventions**

Intervention included in the review:

Any intervention that could be considered predominantly a mobile phone‐based programme (such as text messaging or smartphone apps) for smoking cessation.

Interventions excluded from the review:

Where mobile phones were seen as an adjunct to a predominantly face‐to‐face or Internet programme, such as to remind participants of appointments, or where the effects of the various components of a multi‐faceted programme could not be separated. Telephone counselling was also excluded.

Studies based on comparator were not excluded, but grouped by comparators in the analyses.

**Types of outcome measures**

The primary outcome was smoking abstinence at longest follow‐up, and at least six months from baseline. Where multiple measures were available, sustained abstinence to point prevalence abstinence, and biochemically validated results to self‐report were preferred.

There is no obvious risk of adverse events for text messaging or smartphone app interventions, and so the authors did not included this as an outcome in this review.

**Search methods for identification of studies**

For the present update of the review, the authors searched the Cochrane Tobacco Addiction Group's Specialised Register on 29 October 2018 using the terms 'mobile phone', 'cell phone', 'txt', 'pxt', 'sms', or 'mms' in the title, abstract or keyword fields. The Specialised Register includes reports of possible controlled trials of smoking cessation interventions identified from sensitive searches of databases. At the time of the search, the Register included the following results of searches

- Cochrane Central Register of Controlled trials (CENTRAL; 2018, Issue 1)
- MEDLINE (via Ovid, to 26 October 2018)
- Embase (via Ovid, to 28 October 2018)
- PsycINFO (via Ovid; to 22 October 2018)

See the [Cochrane Tobacco Addiction Group website](https://tobacco.cochrane.org/resources/cochrane-tag-specialised-register) for full search strategies and a list of other resources searched. They also searched the World Health Organization International Clinical Trials Registry Platform (WHO ICTRP; [apps.who.int/trialsearch/](http://apps.who.int/trialsearch/)) and [ClinicalTrials.gov](https://clinicaltrials.gov/) trials registers for ongoing or recently completed studies. They searched through the reference lists of identified studies for any additional eligible studies and attempted to contact the authors of ongoing studies.

No restrictions were placed on publication language or date.

**Data collection and analysis**

**Selection of studies**

The Cochrane Tobacco Addiction Group's Information Specialist ran the searches and provided the results. Two review authors independently pre‐screened the titles and abstracts of records identified in duplicate to exclude reports that had no relevance to the topic and to provide a list of potentially relevant citations. A third reviewer resolved any differences in initial screening. Two review authors independently reviewed full‐text manuscripts in duplicate for the final eligibility screen.

Disagreements were resolved by discussion or by obtaining further information through contacting study authors. Reasons for exclusion of studies were recorded in the [Characteristics of excluded studies](https://www.cochranelibrary.com/cdsr/doi/10.1002/14651858.CD006611.pub5/references#CD006611-sec-0109) table. The authors of unpublished, registered studies, which could potentially have been completed, were contacted to determine ongoing status or to request unpublished data.

**Data extraction and management**

The authors extracted the following methodological details from the included study reports and presented them in the [Characteristics of included studies](https://www.cochranelibrary.com/cdsr/doi/10.1002/14651858.CD006611.pub5/references#CD006611-sec-0108) table. Two review authors independently extracted data using the standardised [Covidence](https://www.cochranelibrary.com/cdsr/doi/10.1002/14651858.CD006611.pub5/references#CD006611-bbs2-0114) data extraction form. A third review author provided a review of the quality assessment and a consensus check.

- Funding source
- Authors' declarations of interest
- Country and context of the study
- Study design
- Number of participants
- Age and other relevant recorded characteristics of study participants
- Inclusion criteria
- Exclusion criteria
- Intervention details
- Control details
- Definition of abstinence outcome
- Smoking cessation rates at six months (self‐reported abstinence or biochemically verified abstinence, or both)
- Smoking cessation rates at final follow‐up (if follow‐up greater than six months and where these data were available)

**Assessment of risk of bias in included studies**

Two review authors independently assessed the risk of bias for included studies, based on the guidance of the *Cochrane Handbook for Systematic Reviews of Interventions* ([Higgins 2017](https://www.cochranelibrary.com/cdsr/doi/10.1002/14651858.CD006611.pub5/references#CD006611-bbs2-0118)), and the Cochrane Tobacco Addiction Group. For each study, they assessed the following domains.

- Random sequence generation
- Allocation concealment
- Blinding of outcome assessment
- Incomplete outcome data
- Other sources of bias

Specific 'Risk of bias' guidance developed by the Cochrane Tobacco Addiction Group to assess smoking cessation studies states that performance bias (relating to the blinding of participants and providers) should not be assessed for behavioural interventions, as it is impossible to blind people to these types of interventions. They graded detection bias as low where there was biochemical verification of abstinence, or where abstinence was self‐reported with no difference in face‐to‐face contact between control and intervention arms. They considered bias due to incomplete outcome as low risk where numbers lost to follow‐up were clearly reported for each group, the overall loss was not greater than 50%, and the difference between groups was not greater than 20%, or sensitivity analysis showed that the direction of effect was not sensitive to different imputation methods for loss to follow‐up.

Each review author recorded information in study reports relevant to each relevant domain and then judged each domain as either at low, high, or unclear risk of bias. Disagreements were resolved through discussion with a third review author.

**Measures of treatment effect**

- Smoking cessation rates at six months or longer using the most stringent measure available
- Biochemically verified abstinence, where available

They calculated risk ratios (RR) and 95% confidence intervals (CI) for the smoking cessation outcome for each included study. They calculated outcomes on an intention‐to‐treat basis, including all participants randomised to a trial arm and assuming that participants lost to follow‐up had continued to smoke or relapsed.

**Dealing with missing data**

Followed up with study authors where possible to request for missing data.

**Assessment of heterogeneity**

In order to assess whether it was appropriate to pool studies and conduct meta‐analyses we assessed the characteristics of included studies to identify any clinical or methodological heterogeneity. Where we deemed studies homogeneous enough to be combined meaningfully, we conducted a meta‐analysis, and we assessed statistical heterogeneity using the I^2^ statistic; we deemed an I^2^ value greater than 50% to indicate substantial heterogeneity ([Higgins 2003](https://www.cochranelibrary.com/cdsr/doi/10.1002/14651858.CD006611.pub5/references#CD006611-bbs2-0117)).

**Assessment of reporting biases**

We planned to use funnel plots to assess reporting bias for any comparisons where we identified and analysed abstinence rates from at least 10 studies. Only the 'text messaging versus minimal smoking cessation support' comparison met this criteria in this review; therefore a funnel plot was generated for this comparison only. Funnel plots illustrate the relationship between the effect estimates from individual studies against their size or precision. The greater the degree of asymmetry, the greater the risk of reporting bias.

**Data synthesis**

They conducted meta‐analyses of the included studies, using the Mantel‐Haenszel random‐effects method to pool RRs and 95% CIs calculated for the smoking abstinence outcome, across the following comparisons.

- Text messaging versus minimal smoking cessation support (including standard self‐help materials, as is standard practice in the Cochrane Tobacco Addiction Group)
- Text messaging in addition to another form of smoking cessation support
- Text messaging versus other smoking cessation support
- Higher‐ versus lower‐frequency text messaging
- Smartphone app versus less intensive smoking cessation support

Where studies had multiple intervention arms relevant to a single meta‐analysis, they split control arm data to avoid double‐counting.

**Subgroup analysis and investigation of heterogeneity**

- Split the 'smartphone app versus less intensive smoking cessation support' comparison into two subgroups to reflect the different comparators used across studies; either minimal non‐app smoking cessation support (e.g. self‐help materials, information on existing stop‐smoking services) or a less intensive smartphone app.

**Sensitivity analysis**

- Calculated pooled RRs and 95% CIs for all analyses using complete case data to calculate quit rates. People may drop out of studies for reasons other than still smoking, and these reasons may differ between groups. For example, people who successfully stop smoking may withdraw from receiving an intervention if the text messages remind them of smoking. Therefore, this analysis tests whether assuming that all people lost to follow‐up are smoking (as in the primary analyses of all participants randomised) is potentially biasing the results.
- Removing any studies judged to be at high risk of bias from all comparisons
- Removing the only cluster‐RCT ([Haug 2013](https://www.cochranelibrary.com/cdsr/doi/10.1002/14651858.CD006611.pub5/references#CD006611-bbs2-0016)), as information was not available to adjust for any potential clustering effect
- Removing the two studies carried out in a pregnant ([Abroms 2017](https://www.cochranelibrary.com/cdsr/doi/10.1002/14651858.CD006611.pub5/references#CD006611-bbs2-0002)), or postnatal population ([Yu 2017](https://www.cochranelibrary.com/cdsr/doi/10.1002/14651858.CD006611.pub5/references#CD006611-bbs2-0026)), as these populations differ substantially from those recruited in the other studies.
