# Supplementary material for: The Systematic Development of a Mobile Phone Delivered Text-Messaging Tobacco Cessation Intervention in India
Source: Nicotine Tob Res. 2024 Dec 21;27(9):1616–25. doi: 10.1093/ntr/ntae306 (PMC12370465; doi:10.1093/ntr/ntae306)
Supplement: ntae306_suppl_Supplementary_Appendices [file ntae306_suppl_supplementary_appendices.zip › ntae306_suppl_Supplementary_Appendix_2.docx]

**Appendix 2 :** Qualitative interview guides used to conduct the semi-structured in-depth interviews with users of tobacco and tobacco cessation experts.

**Alt text:** The text below lists out all the questions of the qualitative interview guide which the trained qualitative researchers used to conduct semi-structured in-depth interviews with users of tobacco and tobacco cessation experts.

**2.1 Semi-structured in-depth Interview guide for intended participants of the intervention – Formative Phase**

**Introduction**

(To be conducted after administering the Socio- demographic form)

Now, I will be asking you a few more questions to hear more from you.

*Tape recording information:* I will be recording our conversation so as to ensure I am capturing all our discussion points while also paying complete attention to you. The recording will only be available to our research team for the purposes of the project. No other individual will have access to the same and utmost care will be taken to keep your personal details confidential. At any point, if you feel uncomfortable with being recorded, please let me know and we can stop recording.

Can we begin?

**I. Patterns of Tobacco usage**

**1. Can you tell me how and/or why you have come to use tobacco?**

**Probes**:

- At what **age** did you begin using tobacco?
- How long have you been using tobacco for?
- Were you **introduced to tobacco** by a friend/ family member/ relative/ colleague?
- What **prompted you** to try tobacco? (social event, peer pressure, novelty and so on)

**2. Generally, what prompts you to use tobacco?**

*Note: Triggers could be tied to internal or external factors such as company in social settings, or habits such as after meals and so on. There* ***are several reasons*** *why an individual may be using tobacco. These reasons can be classified into the following areas or domains.*

*1. Physical/ biological,*

*2. Emotional/psychological,*

*3. Social/ behavioural/ habitual*

*Explore reasons across the three main areas of dependence*

**Probes:**

- ***Habitual dependence:***

Do you feel the need to use tobacco in association with certain activities such as drinking tea, cutting vegetables?

Do you feel tempted to use tobacco at certain times of the day such as after dinner, while watching television or as soon as you wake up in the morning (could be related to bowel movements too)?

- ***Biological dependence:***

Do you feel irritated or restless if you do not use tobacco?

Do you experience headaches or any other kind of pain or aches if you do not use tobacco?

- ***Emotional dependence:***

Do you feel tempted to use tobacco when you are sad, stressed or upset? (in response to any negative emotion)

Do you feel tempted to use tobacco when you are happy or excited? (in response to any positive emotion)

**II. Impact of Tobacco Usage**

**1. ‘How has using tobacco affected your life?’ Or ‘What has changed in your life after you started using tobacco?’**

*Note: Explore effects in the areas of health, occupational functioning and social functioning using the probes given below.*

**Probes**:

- ***Health***

***Have you faced issues such as (refer to the points below) after onset of tobacco use?***

Health problems (symptoms) such as shortness of breath, headaches, dizziness, dental problems such as bleeding gums or tooth decay, eye sight related issues, and improved alertness or pain relief and so on following onset of tobacco use

Long term health issues such as heart diseases, cancer, tuberculosis and so on following onset of tobacco use.

Caused or aggravated existing mental health issues such as increased anxiety, restlessness, decreased concentration, experience of stress and so on.

- ***Occupational functioning***

Have you had any changes in performance at work due to tobacco? Or loss of job? What were the circumstances that caused this?

Were there any resulting hardships (e.g. financial difficulties, problems in relationships with colleages)?

- ***Economic conditions***

Can you tell us if you’ve had any **financial hardship** related to tobacco? Any changes in your economic resources? Do you spend on tobacco instead of other important items? Does this cause troubles in your relationships?

- ***Social functioning:***

Can you tell us a little about if and how using tobacco has affected your social interactions and experiences?

Do you notice any changes in your family life in terms of your relationship with family members and/ or the family environment since the onset of tobacco use?

**III Treatment seeking**

**1. Have you tried to reduce or quit tobacco in the past? If yes, how did you go about it? *(self-help, counsellor, doctor)***

*Note: Capture experiences with practitioners, doctors, counsellors, any app/ helpline/ SMS service.*

**Probes:**

Have you tried to **cut down** on tobacco usage in the past?

Have you been able to **completely quit** tobacco use at any point in time?

Have you received any treatment in the past for tobacco usage? If yes, could you share details of the nature of treatment and your experience with the same?

**2. *If yes to question 1,* What helped you cut down/ remain tobacco free? What do you think would have helped your attempt to cut down/ stay tobacco free?**

**3. *If yes to question 1:* Has the quit/ reduce attempt resulted in any changes in your tobacco usage over time (increase or decrease)?**

**Probe: Switching between substances**

**4. *If yes to question 1:* What were some of the challenges you faced in attempting to quit tobacco?**

**IV. Proposed Intervention**

**1. What are some of the topics that you would like to see in a tobacco cessation intervention?**

**Following are some of the areas that we plan to cover. Please indicate three that you think are most important according to you.**

A. Information on tobacco usage and statistics - generic

B. Habit breaking/ behaviour modification - Coping with urges & handling triggers

C. Problem solving skills

D. Quit planning & tips to quit

E. Handling nicotine withdrawal

F. Contact details of quit clinics and other important contact information

G. Other areas:

**2. *Nature of content:* What kind of information do you think a tobacco cessation treatment should focus on?**

**Probes:**

- *Motivational/ informational/ interventional messages:* Would you rather receive motivational messages encouraging you to quit and to stay quit? Or, would you prefer exercises and activities that help you develop coping mechanisms, strategies for overcoming addiction and so on?

- *Tailored/ Generic:* Would you be more interested in engaging with information that is tailored to your needs and experience? Or, would you prefer generic information on tobacco and quitting?

**3. *Frequency and length of treatment:* According to you, how long should a treatment program of this nature be?**

**Probes:**

Keeping your daily schedule in mind, how much time would you have to engage with a treatment for tobacco cessation in a month/ week?

What would be your preferred days of engagement and preferred time of the day? (weekdays/ weekends; late evening, early morning or through the day)

**V. Technology & interface**

**1. What is your experience with the SMS feature on your mobile phone?**

**Probe:**

Do you regularly send or receive a SMS?

**2. Have you received pre-recorded voice calls on your phone? What was your experience? Do you think receiving pre-recorded voice calls on important information is helpful/ useful?**

**3. *Interactivity:* Would you prefer messages that require inputs/ responses from your end or would you rather receive informational texts that do not require you to acknowledge/ respond in any way?**

*Note: Share an example of a commercial message that requires user input.*

**4. *Peer Vs Expert voice:* If you were to receive a pre-recorded voice call, would you be more interested if the message came from someone like a peer of your age encouraging you to quit or would you be more interested if an expert (e.g. a doctor)/a popular role model (e.g. an actor) gave you tobacco-related information?**

**5. What do you think about using mobile phones to deliver a tobacco cessation treatment?**

**Probes:**

If you were to receive a tobacco cessation treatment over SMS, what would your thoughts and concerns be?

What do you think of the impact of a SMS delivered tobacco cessation treatment?

We have now covered all the questions I had for you. Is there anything else that you would like to discuss? Do you have any questions for me? *Probe for queries/ concerns and answer if any.*

Thank you once again for taking the time to talk to me today. Your opinions and suggestions will be immensely valuable for us in developing this program.

**2.2 Semi-structure in-depth interview guide for tobacco cessation experts**

**Aims:**

- To understand characteristics of tobacco use in the Indian context (frequency of smoked vs smokeless tobacco use, uptake of intervention among the two groups and so on)
- To understand existing trends in treatment to inform options for care
- To understand experts’ perspectives on requisite treatment content and methodology (factors causing relapse, factors preventing adequate uptake of interventions among users)

**I. Patterns of tobacco use**

1. From your experience, what are some of the differences in the nature of tobacco use among smoked and smokeless tobacco users?

2. How do you define problematic tobacco use?

**Probe:**

- Do you distinguish tobacco use into different types based on extent of use?

3. Have you used any specific assessment tools or methods to determine whether an individual is using tobacco in a problematic manner? If yes, please can you describe the tool/method in more detail.

**II. Tobacco cessation treatment**

4. According to you, what are some of the topics (or areas) that must be covered in a tobacco cessation intervention?

**Probe:**

- Based on your experience, do you think interventions should vary for smoked and smokeless tobacco use? Please indicate the components of treatment that are more relevant to one than the other.

5. Do you refer to a specific model of drug/ tobacco dependence in planning treatment?

7. As per your experience, what are the usual reasons for relapse among tobacco users?

**Probe:**

- What is the difference in relapse patterns among smoked or smokeless tobacco users?

8. What are some of the relapse prevention strategies you employ?

**Probe:**

- What are the differences in relapse management strategies for smoked and smokeless tobacco users?

9. What are some of the challenges you experience in delivering tobacco cessation treatments?

**III. Factors affecting treatment uptake**

9. Based on your experience, what are the differences in the uptake of treatment among smoked and smokeless tobacco users?

10. What are some factors that motivate users to seek (and/or continue) treatment?

**Probe**: As per your experience, at what stage do tobacco users seek treatment? Are there any differences between smoked and smokeless tobacco users?

11. According to you, what are some factors that are barriers to seeking treatment and/or continuing treatment among tobacco users?

**V. Mode of treatment delivery – Technology and interface**

12. What are your thoughts on using mobile technology for tobacco cessation treatment?

13. If mobile technology were to be used to deliver a tobacco cessation treatment, which according to you would be an ideal platform for the same?

**Probe**: e.g. SMS, IVR, WhatsApp, FB Messenger

14. What are some of the potential drawbacks that you foresee in using mobile phone technology, specifically SMS and IVR, in delivering tobacco cessation treatment?
